# Using design of experiments (DoE) to optimize performance and stability of biomimetic cell membrane-coated nanostructures for cancer therapy

**DOI:** 10.3389/fbioe.2023.1120179

**Published:** 2023-02-02

**Authors:** Natália Noronha Ferreira, Renata Rank Miranda, Natália Sanchez Moreno, Paula Maria Pincela Lins, Celisnolia Morais Leite, Ana Elisa Tognoli Leite, Thales Rafael Machado, Thaís Regiani Cataldi, Carlos Alberto Labate, Rui Manuel Reis, Valtencir Zucolotto

**Affiliations:** ^1^ Nanomedicine and Nanotoxicology Group, Physics Institute of São Carlos, São Paulo University, São Carlos, Brazil; ^2^ Max Feffer Laboratory of Plant Genetics, Department of Genetics, ESALQ, University of São Paulo, Piracicaba, Brazil; ^3^ Molecular Oncology Research Center, Barretos Cancer Hospital, Barretos, Brazil; ^4^ Life and Health Sciences Research Institute (ICVS), School of Medicine, University of Minho, Braga, Portugal; ^5^ ICVS/3B’s—PT Government Associate Laboratory, Braga, Portugal

**Keywords:** cell membrane coating technology, mass spectrometry-based proteomics, biomimetic systems, stability, design of experiment-DoE, homotypic recognition, glioblastoma, temozolomide drug

## Abstract

**Introduction:** Cell membrane-covered biomimetic nanosystems have allowed the development of homologous nanostructures to bestow nanoparticles with enhanced biointerfacing capabilities. The stability of these structures, however, still represents a challenge for the scientific community. This study is aimed at developing and optimizing cell derived membrane-coated nanostructures upon applying design of experiments (DoE) to improve the therapeutic index by homotypic targeting in cancer cells.

**Methods:** Important physicochemical features of the extracted cell membrane from tumoral cells were assessed by mass spectrometry-based proteomics. PLGA-based nanoparticles encapsulating temozolomide (TMZ NPs) were successfully developed. The coating technology applying the isolated U251 cell membrane (MB) was optimized using a fractional two-level three-factor factorial design. All the formulation runs were systematically characterized regarding their diameter, polydispersity index (PDI), and zeta potential (ZP). Experimental conditions generated by DoE were also subjected to morphological studies using negative-staining transmission electron microscopy (TEM). Its short-time stability was also assessed. MicroRaman and Fourier-Transform Infrared (FTIR) spectroscopies and Confocal microscopy were used as characterization techniques for evaluating the NP-MB nanostructures. Internalization studies were carried out to evaluate the homotypic targeting ability.

**Results and Discussion:** The results have shown that nearly 80% of plasma membrane proteins were retained in the cell membrane vesicles after the isolation process, including key proteins to the homotypic binding. DoE analysis considering acquired TEM images reveals that condition run five should be the best-optimized procedure to produce the biomimetic cell-derived membrane-coated nanostructure (NP-MB). Storage stability for at least two weeks of the biomimetic system is expected once the original characteristics of diameter, PDI, and ZP, were maintained. Raman, FTIR, and confocal characterization results have shown the successful encapsulation of TMZ drug and provided evidence of the effective coating applying the MB. Cell internalization studies corroborate the proteomic data indicating that the optimized NP-MB achieved specific targeting of homotypic tumor cells. The structure should retain the complex biological functions of U251 natural cell membranes while exhibiting physicochemical properties suitable for effective homotypic recognition.

**Conclusion:** Together, these findings provide coverage and a deeper understanding regarding the dynamics around extracted cell membrane and polymeric nanostructures interactions and an in-depth insight into the cell membrane coating technology and the development of optimized biomimetic and bioinspired nanostructured systems.

## 1 Introduction

Nanotechnology has overcome important shortcomings over conventional cancer therapy, providing significant features for a precise and early diagnosis, as well as for remarkable clinical outcomes in therapy (Fang et al., 2019; Guo et al., 2021). One of its key features is the possibility of encapsulating antineoplastic drugs into delivery systems. These nanostructures offer significant advantages to cancer therapy as they may overcome long-term issues such as poor drug solubility, drug metabolism, systemic half-life, and undesirable side effects. The nanoplatform should also significantly benefit from the Enhanced Permeability and Retention (EPR) effect, which leads to increased drug accumulation into the tumor stroma (Kumari et al., 2015; Patra et al., 2018). The EPR effect may be further enhanced by decorating the nanostructure surface with ligands (i.e., proteins, peptides, nucleic acids) (Rawal and Patel, 2019) able to interact with specific molecules overexpressed in cancerous cells, resulting in an increased efficacy (Yu et al., 2016). More recently, sophisticated bioinspired and biomimetic targeting techniques applying isolated natural membranes of cancer cells have been explored to intensify targetability. Those systems have also been used in the so-called “nano-immunotherapies” to induce immune responses against metastatic tumors (Comparetti et al., 2022).

Firstly introduced by Hu and co-workers (Hu et al., 2011) this novel bioinspired strategy, able to provide nanoparticle accumulation in the tumor microregion and particle camouflage, is composed of a specific functionalization that applies coating with isolated natural cell membranes originating biomimetic nanosystems (Marangoni et al., 2019; Xiang et al., 2021). Isolated cell membranes bestow not only important biological features of natural cells but also provide convenient properties for efficient drug delivery, such as prolonged blood circulation and lower immunogenic potential compared with conventional nanoscale platforms (Luk et al., 2014). Additionally, the cell membrane coating technology may significantly improve the NP stability during transportation and storage (Xiang et al., 2021). Especially for cancer treatment, the biomimetic nanosystems provide self-recognition between expressed proteins, a remarkable feature with the potential to afford immune evasion and to effectively accumulate at the tumoral site, enabling homotypic targeting and effective interaction with the complex biological tumor microenvironment, a property associated to which their plasma membrane cell-cell adhesion proteins (Chen et al., 2020a; Zhang et al., 2022).

Whilst a considerable number of publications have already been published applying cell-derived membrane-coated nanostructures, there are still many challenges to address in an attempt to translate this technology into clinical applications (Xiang et al., 2021). Therefore, studies covering an extensive discussion regarding the design in terms of variables, stability, and physicochemical characterization are of great interest to the scientific community.

It is well established that transmission electron microscopy (TEM) and dynamic light scattering (DLS) provide essential information regarding the successful coating of the tumoral cell membrane into the polymeric nanostructured core. However, further verification of the biological composition and activity of the biomimetic structure is needed to ensure their organic features. The detailed analysis of cell membrane protein profiles and biomarker reservations is mandatory for the resultant nanostructure to inherit the special biological functions after the coating procedure (Chen et al., 2020a).

Here, we used associated techniques and statistical tools to optimize the development of biomimetic PLGA-based nanoparticles coated with U251 (GBM) cell membranes to increase the specificity of temozolomide (TMZ) drug to different glioma cell lines. First, mass spectrometry-based proteomics was applied to identify the isolated GBM cell membrane proteins, their recovery and stability aspects. The statistical design of experiments (DoE) was used to optimize the development of biomimetic nanoparticles coated with U251 cell membranes to increase the specificity by homotypic recognition of glioma cell lines. To our knowledge, this is the first time DoE has been applied to optimize the coating of nanosystems using isolated cell membranes, correlating the physicochemical aspects with the morphological images from Transmission Electron Microscopy (TEM). Vibrational spectroscopies and confocal microscopy were further explored to provide evidences of the membrane coating features. Flow cytometry analysis was applied to verify targeting specificity.

Our results revealed that the optimization of the biomimetic and bioinspired nanostructures with tailored properties designed for TMZ encapsulation and homotopic recognition was successfully achieved by applying statistical design of experiments (DoE) together with important physicochemical techniques.

## 2 Methods

### 2.1 Cell line and cell culture

Comercial human glioblastoma cells (U251 and U87), and primary glioblastoma cell line (HCB 151) derived from surgical biopsies obtained in the Neurosurgery Department of Barretos Cancer Hospital (Martinho et al., 2013; Teixeira et al., 2022) (Sao Paulo, Brazil), acquired by the local ethics committee approval and the patient’s consent agreement, were kindly donated from Dr. Rui Reis, Barretos Cancer Hospital. Cell authentication was performed through short tandem repeat (STR) DNA typing, according to the International Reference Standard for Authentication of Human Cell Lines. Human Dermal Fibroblast—neonatal (HDFn) cell line was obtained from Sigma Aldrich (São Paulo, Brazil) and used as a non-malignant cell line. All cell lines were grown in Dulbecco’s Modified Eagle’s medium (DMEM; Vitrocell^®^ Embriolife) supplemented with 10% fetal bovine serum (FBS), gentamicin sulfate (0.05 mg/mL), and amphotericin B (25 μg/mL), L-glutamine (0.584 mg/mL) at 37°C in a humidified incubator, with an atmosphere of 95% air and 5% CO_2_.

### 2.2 Development of PLGA-based nanoparticles (NP) using double emulsion solvent evaporation technique

Poly (D, L-lactide coglycolide) (PLGA) NP was produced using the double emulsion (W1/O/W2) method with modifications, initially proposed by Ramalho and co-workers. Briefly, 1 mg of TMZ drug (Sigma Aldrich, Brazil) and 5 mg of (PLGA 85:15) (Lactel Biodegradable Polymers) were dissolved in 1 mL dichloromethane (DCM) and 1 mL acetone, respectively, composing a homogeneous 2 mL organic phase. 500 µL of the internal aqueous phase (polyvinyl alcohol—PVA 2%) (Sigma Aldrich, Brazil) was sonicated into the organic phase at 30 W for 1 min in an ice bath. Afterward, the resultant milky solution was sonicated in the external aqueous phase (2 mL–0.02% PVA) at 30 W for 1.5 min in an ice bath. Finally, the NP dispersion was left under magnetic stirring for organic solvent evaporation and washed using Amicon^®^ 100-kDa cut-off for further characterization. Blank NP was also produced for comparative purposes. The encapsulation efficiency analyses (EE%) of TMZ into PLGA nanoparticles were performed applying validated reverse-phase high-performance liquid chromatography (RP-HPLC) methodology adapted from Michels and co-workers (Michels et al., 2019) with standard analytical curve built-in acetic acid 0.5% (y = 74177x + 1360,6; *r* ^2^ = 1,00).

For internalization studies, 200 µL of 1 mg/mL 3,3′-Dioctadecyloxacarbocyanine perchlorate solution (DiO, Sigma-Aldrich) in DCM was added to the organic phase before the sonication procedure. In this case, NP-DiO was further dialyzed overnight against PBS using dialysis tubing cellulose 12 kDa cut-off (Sigma Aldrich, United States).

### 2.3 Extraction of U251 glioblastoma cell membrane (MB)

U251 tumor cell membranes were isolated and purified following an extensive protocol previously proposed by Lund et al., 2009, modified by Marangoni and co-workers, 2019 (Marangoni et al., 2019). Briefly, once cells reached approximately 80% confluence, culture media was replaced by ice-cold phosphate buffer saline 1× (PBS), and cells were detached from the cell culture flask with the aid of a cell scraper. Then, the cell dispersion was washed three times with ice-cold PBS and incubated on ice with hypotonic and gradient buffers, following extraction cycles using a homogenizer. The disrupted cell dispersion was centrifuged at 10.000 g for 15 min to remove debris and non disrupted cells, and the resultant supernatant was further centrifuged at 100.000 g for 2 h, at 4°C. The pelleted cell membranes were resuspended with PBS plus protease inhibitors and kept at −80°C until further use.

### 2.4 Physico-chemical characterization of produced NP and MB

The physicochemical features of the NP and extracted membrane, such as mean diameter, size distribution (PDI), and zeta potential (ZP) values were assessed by Dynamic Light Scattering (DLS) using photon correlation spectroscopy, a wavelength of 633 nm at 25°C, detection angle of 90°, and the electrophoretic mobility in a Zetasizer Nano ZS (Malvern Instruments, Malvern, United Kingdom) equipment. Concentration and size distribution were also recorded using nanoparticle tracking analysis (NTA). The analyses were carried out in a NanoSight NS300 (Malvern Instruments, Worcestershire, United Kingdom) equipped with a sample chamber and a 532 nm laser. The NP and cell membrane were diluted using ultrapure water and injected into the sample chamber. For DiO-loaded NP characterization, a fluorescence filter was applied for data acquisition. All samples were diluted in PBS, and the results are presented as the average of three independent measurements (n = 3) and their standard deviation (SD). The quantitative determination of protein content in the extracted U251 cell membrane was performed by using Bradford Assay (Thermo Scientific™ Pierce™) following the instructions described in the manufacturer’s protocol applying a calibration curve using known concentrations of bovine serum albumin (BSA).

### 2.5 Evaluation of isolated MB integrity and composition by mass spectrometry-based proteomics

#### 2.5.1 MB sample preparation for MS-based proteomics analysis

U251 cultured cell, freshly isolated cell membranes (MB), and isolated MB stored in PBS with protease inhibitors (Sigma Aldrich, Brazil) at −80°C (during 6 months) were resuspended in lysis buffer (7 M urea, 2 M thiourea, 10 mM DTT, and 0.01% Triton X-100) at room temperature for 2 h. Following, the lysis buffer was removed using Amicon^®^ Ultra 0.5 3KD (Millipore). The protein content was quantified using QuantiPro™ BCA Assay Kit (Sigma Aldrich, Brazil) and NanoDrop One (Thermo Scientific, United States). To confirm protein quantification and to check the protein banding pattern, a 10% SDS-Page gel analysis of the samples was performed by applying 10 ug of BSA as a standard protein sample. All samples were mixed to sample buffer (0.5 M Tris-HCl, 87% glycerol, β-mercaptoethanol, 1% bromophenol blue, 20% SDS) in a 1:1 ratio and applied to the gels a final concentration of 20 µg. The gel run was performed at 50 v for 4.5 h. After the run, the gel was removed from the glass plates and added to a fixative solution (50% methanol and 7% acetic acid) for 1 h, followed by washing with Milli-Q water and left overnight in the GelCode Blue stain.

Samples (10 µg) were incubated with RapiGest™ SF Surfactant (Waters, United States) for 15 min, 80°C, and 100 mM dithiothreitol (DTT) for 30 min. Alkylation was performed using 300 mM iodoacetamide (IAA) for 30 min in the dark, following digestion with trypsin (1:100 w/w trypsin: protein) overnight, at room temperature. Finally, the peptides were acidified using 1% v/v formic acid to stop the trypsin digestion and lyophilized before desalting (Rank Miranda et al., 2020). Samples were resuspended in 0.1% v/v trifluoroacetic acid (TFA) and desalted using ZipTipC18 Pipette Tip with C18 Resin (Sigma Aldrich, Brazil) following the manufacturer’s instructions.

#### 2.5.2 Sample fractionation and mass spectrometry (MS) analysis

The mass spectrometry analysis were carried out using the nanoElute nanoflow chromatographic system (Bruker Daltonics, Bremen, Germany) coupled online to a hybrid trapped ion mobility spectrometry-quadrupole mass spectrometer time-of-flight mass spectrometer-timsTof Pro (Bruker Daltonics). An aliquot (1 µL) of the sample, equivalent to 200 ng of peptides, was injected into an Aurora 2 C18 trap column (1.6µm, 250 mm × 75 µm) (ionOpticks, Australia). A typical RP gradient (Solvent A: 0.1% AF, 99.9% H_2_O MilliQ; Solvent B: 0.1% AF, 99.9% CH_3_CN) was established in a liquid chromatography nanoflow system and separated at a flow rate of 400 nL.min^− 1^. The column temperature was maintained at 50°C. The chromatographic run was 120 min (2%–15% Solvent B for 60 min; raised to 25% at 90 min; raised to 37% at 100 min; raised to 95% at 110 min and finally 95% for 10 min for column washing).

The column was coupled, online, to a timsTOF-Pro with a CaptiveSpray ion source, (Bruker Daltonik GmbH). The temperature of the capillary ion transfer line was set to 180°C. Ion accumulation for 123 ms and separation by mobility were obtained with an input potential ramp from −160 V to −20 V within 123 s. During the acquisition, to enable the PASEF method, i.e., the accumulation parallel to the fragmentation of the ions, the precursor m/z and mobility information was first derived from a Tims-MS full scan experiment, with an m/z range of 100–1700. Monocharged precursors were excluded for their position in the m/z-ion plane of mobility, and precursors that reached the target value of 20,000 a.u. were dynamically excluded for 0.4 min. The operational mode of the TIMS-ToF, MS and PASEF were controlled and synchronized with the aid of the instrumental control software OtofControl 5.1 by Bruker Daltonik.

#### 2.5.3 Processing parameters and search in public databases

Data processing, protein identification and relative quantification analyzes were performed using the MaxQuant Software (https://www.maxquant.org/). Processing parameters included: carbamidomethylation of cysteine as a fixed amino acid modification. Methionine oxidation and N-terminal acetylation were considered variable variations. Trypsin was used as a proteolytic enzyme, with a maximum of 2 possible cleavage errors. The ion mass shift tolerance for peptides and fragments was adjusted to 20 ppm and 0.05 Da, respectively.

A maximum false positive rate (FDR) of 1% was used for peptide and protein identification, considering at least one single peptide for protein identification as a criterion. All the proteins were identified with a confidence level ≥95% using the MaxQuant algorithm and searching within the revised Human Uniprot database (https://www.uniprot.org/uniprotkb?facets=reviewed%3Atrue%2Cmodel_organism%A9606&query=Human), with 20,401 sequences (Macron et al., 2020). Bioinformatic analysis was performed based on UniProt and Gene Onthology (GO) (http://www.uniprot.org/and http://www.geneontology.org/, respectively) information and manual literature mining. The mass spectrometry proteomics data have been deposited to the ProteomeXchange consortium *via* the PRIDE partner repository with the dataset identifier PXD039270.

### 2.6 Design of experiments (DoE) to optimize the development of cell-derived membrane-coated nanostructures (NP-MB)

A fractional two levels three-factor design of experiment (DoE) was applied to optimize cell-derived membrane-coated nanostructures development. The use of DoE enables the operation of a minimum number of experiments to evaluate the effect of the following variables: NP:MB ratio (X_1_), method of coating procedure (X_2_), and time/cycles (X_3_) applied on the responses of size (S), zeta potential (ZP), and polydispersity index (PDI). The lower (−1) and higher values (+1) selected for each variable were chosen according to pre-formulation studies and literature research (Ming, 2011; Jin et al., 2019; Chen et al., 2020b; Cui et al., 2020; Wang et al., 2020). The codified variable values under low and high levels are represented by (−1) and (+1) as summarized in Table 1.

**TABLE 1:** Variables with respective coded levels of the fractional factorial design.

Levels	NP: Membrane ratio	Method	Time/Cycles (min)
−1	1:1	Extrusion	10
1	1:5	Sonication bath	20

A total of 8 formulation runs were generated, as shown in Table 2. In the case of mechanical extrusion, developed PLGA NP was extruded with the isolated membrane through 200 nm cellulose acetate membranes (Whatman, United States) for 10/20 cycles times using Avanti Mini-Extruder (Avanti Polar Lipids, United States). The sonication bath method included the NP and MB samples sonication separately (80 W potency and 37 kHz frequency; at 10°C for 10 min) following the same process for an extra 10/20 min where NP was added to MB.

**TABLE 2:** Physicochemical aspects of developed blank NP, NP, and isolated U251 membrane (MB). Size, PDI, and ZP data from DLS analyses; mean and particle concentration from NTA analyses. Bradford assay was performed to record protein content. Data represent the average of at least 3 measurements (n = 3) and standard deviation.

Sample	EE%	Dynamic light scattering (DLS)			Nanotracking analysis (NTA)		Bradford assay
		Size (nm)	PDI	ZP (Mv)	Mean (nm)	Concentration (particles/mL)	Protein concentration (mg/mL)
Blank NP	nd	178 ± 23	0.07 ± 0.02	−20 ± 2	193 ± 17	2 × 10^11	nd
NP	55 ± 15	180 ± 5	0.12 ± 0.02	−17 ± 3	186 ± 10	3 × 10^11	nd
MB	nd	389 ± 70	0.59 ± 0.06	−27 ± 3	239 ± 8	1 × 10^11	0.17 ± 0.06

The physicochemical characterization was performed for size, PDI, and ZP values using the DLS and sample dilution in PBS (12.5x). The analysis of NP, MB, and the physical mixture between NP and MB (where no sonication or extrusion was applied) was also performed for comparative purposes. StatSoft Inc. (2004) STATISTICA (data analysis software system) version 7 was used to perform the regression analyses and interaction calculation among independent factors. Statistical significance was set considering a 95% confidence level.

#### 2.6.1 Physicochemical characterization of experimental conditions generated by DoE

For a deep characterization of the experimental conditions (8 runs), all samples were additionally characterized using Nanoparticle Tracking Analysis (NTA) in a NanoSight NS300 (Malvern Instruments, Worcestershire, United Kingdom), equipped with a sample chamber and a 532 nm laser, camera level 11/12, and 80 ± 30 particles per frame for mean size, and concentration. The NP-MB nanostructures were diluted using PBS (100x). Samples were stored at 8°C and the short-time stability (15 days) of those systems was checked by the analysis of size, PDI, and ZP using the Zetasizer NanoZS (Malvern Instruments, Malvern, United Kingdom), as described above.

Microscopic analyses were carried out using negative-staining Transmission Electron Microscopy (TEM) JEM-2100-JEOL 200 with a LaB6 source operating at an acceleration voltage of 200 kV. Samples were drop-cast onto a 400 Cu mesh carbon film TEM grid and stained using uranyl acetate solution 2% (w/v). Excess samples and uranyl acetate were drained using filter paper.

### 2.7 Vibrational spectroscopies and confocal characterization of optimized MB-NP nanostructure

The Raman spectroscopy can be applied to the analysis of organic and biomimetic materials such as polymeric nanoparticles and cell membranes by measuring the non-elastic scattering of photons, i.e., Raman scattering, provided by the laser irradiation which can give the fingerprint vibrational spectra of target molecules (Xing et al., 2020). Therefore, attempting to study the NP constituents and possibly the coating procedure applied isolated MB, Raman spectra of blank NP, NP (TMZ loaded NP), NP-MB, and respective controls were collected in an *Via*™ Raman confocal microscope (Renishaw, United Kingdom) with a 532 nm laser (power of 50%) using a ×20 objective and a grating of 1800 lines/mm. The samples were drop-cast onto a microscope slide substrate and allowed to dry under a reduced atmosphere in desiccator. Single spectrum measurements were recorded in the 1200–3800 cm^−1^ range with 5 s acquisition and 30 accumulations in each spectra.

Fourier-Transformed Infrared measurements were conducted using silicon substrates (P-type/boron-doped silicon, Sigma-Aldrich) previously cleaned with isopropanol, ethanol, acetone, and water in an ultrasound bath (Marangoni et al., 2019). The samples were drop-cast onto a silicon substrate and allowed to dry under a reduced atmosphere in desiccator (Maria et al., 2022). The spectra were collected in transmittance mode using an Infrared spectrometer VERTEX 70, (Bruker, United States), with 128 scans per sample with 4 cm^−1^ resolution from 4,000 to 400 cm^−1^.

To additionally check whether the coating procedure was successfully achieved, the prepared samples were analyzed *via* confocal laser scanning microscopy (CLSM) in a LSM 900 confocal microscope (Zeiss, Germany). 3,3′-dioctadecyloxacarbocyanine perchlorate solution (DiO, Sigma Aldrich) and the CellMask™ Deep Red plasma membrane stain (Life Technologies) was used to label the NP and MB, respectively. 5 µL of fluorescent NP, MB, NP-MB, and physically NP + MB (5 × 10^9^ particles/mL), were dripped on top of microscope glass slides and images were recorded with a 40x/1.3 oil objective, keeping the same parameters (e.g., laser power, brightness and contrast adjustments) (Dehaini et al., 2017). Samples were excited at λ_exc_ = 488 nm and 640 nm to collect the green (λ_em_ = 521 nm) and red (λ_em_ = 670 nm) emissions departing from NP and MB fluorescent labels, respectively. Images recorded from NP, MB, NP-MB, and physical mixture (NP + MB) were compared in an attempt to identify visual evidence of the coating process. For this purpose, the pixels on those images (n = 5) were categorized by color and then counted. Firstly the Dio and Cell Mask images were analyzed to get a baseline of what would be considered a red or green pixel. Given those values, of −3° ± 1° for the red pixels and hue of 128° ± 8° for the green pixels, any pixel that the hue value (h) satisfies −3° < h < 120° can be considered yellow. To perform this task, a script written in the Python programming language with the OpenCV Library was used.

### 2.8 Internalization studies and evaluation of homotypic targeting ability of MB-NP nanostructure

Cell internalization assays were carried out by analyzing the NP and NP-MB uptake by the U87, U251, HCB151, and HDFn cell lines. Fluorescent NP and NP-MB were produced by the addition of 200 uL of 2 mg/mL DiO in dichloromethane to the organic phase before the NP sonication procedure above described. Cells were seeded onto 12 well plates at a density of 1 × 10^5^ cells/well and allowed to adhere overnight. Afterward, cells were incubated either with NP or NP-MB, at 5 × 10^10^ particles/mL, for 8 h. After incubation, the culture medium was removed, and the cells were washed 3 times with PBS, harvested (0.25% trypsin, 0.02% EDTA in PBS, pH 7.2), and pelleted in a complete culture medium (1,000 *g*, 5 min). The pellets were washed by centrifugation (500 *g* for 10 min, 4°C) and resuspended with 0.5% BSA-Isoton solution. The cell suspension was analyzed by flow cytometry on a FACS Calibur (BD Biosciences) using untreated cells as controls. Three independent biological replicates (n = 3) were performed for each cell line, and data were processed using FlowJo software. Statistical analysis was performed using GraphPad Prism Software Version 8.0 (GraphPad Software Inc.).

## 3 Statistical analysis

Statistical analysis was carried out using GraphPad Prism Software Version 8.0 (GraphPad Software Inc.). Differences between experimental groups were compared using a one-way analysis of variance and the medium was compared using the Tukey *post hoc* test. The results are shown as a mean ± standard deviation from at least three independent experiments (n = 3). Differences are considered significant at **p < 0.05 and ***p < 0.01.

## 4 Results and discussion

### 4.1 Development and physicochemical characterization of NP and isolated MB

The solvent evaporation technique has been the most used methodology for TMZ drug encapsulation into polymeric nanostructures (Jain and Jain, 2013; Ananta et al., 2016). However, considering drug physicochemical aspects such as aqueous solubility, logP, its low molecular weight (194.15 g/mol), and the conventional difficulty of encapsulation into PLGA cores, the acquisition of high rates of EE% strongly depends on TMZ supersaturation in the aqueous phase (Ling et al., 2012; Ananta et al., 2016). Therefore, we have transposed the single emulsion and drug saturation alternatives into the use of double emulsion solvent evaporation applying a solvent mixture that could enable higher amounts of drug loading (Ananta et al., 2016; de Oliveira Junior et al., 2020). Table 2 shows that the adopted methodology provided nanostructures of about 180 nm, with a monodisperse profile (0.07–0.12), ZP of about −20 mV, and 50 ± 15 of TMZ EE%. NTA data also corroborates DLS results showing a mean size of around 190 nm. Particle concentration was similar for blank NP and TMZ-loaded NP.

The extracted cancer cell membranes following previously described protocols validated by us (Marangoni et al., 2019; Comparetti et al., 2020), showed mean size and PDI values significantly higher than produced nanostructures while ZP showed a stable negative charge (Table 2). NTA data also displayed increased mean size and reduced concentration compared with blank NP and NP (TMZ loaded NP). Quantitative Bradford assay revealed that the total protein concentration of extracted cell membrane was 0.17 ± 0.06 mg/mL (Table 2).

The previous characterization of the NP and the MB constitutes an important early step for the design of membrane cell-derived membrane-coated nanostructures. These particular characteristics can provide a deeper analysis, from a colloidal perspective, of the engineering of biomimetic and bioinspired platforms (Luk et al., 2014). To date, the measurement of particle size and ZP of both nanostructures can give an idea regarding the coating procedure in terms of stability, particle membrane ratio applied, and estimation for complete coverage. Recorded mean size values for isolated membrane (MB) proved to be higher than the sizes of the NPs, leading us to investigate membrane-to-polymer ratios equal to or greater than 1 (NP: membrane ≥1).

The electrostatic interactions are the main driving force to promote membrane interactions with a nanoparticle substrate (Mornet et al., 2005). In this regard, Luk et al. (2014) and co-workers have exploited the interfacial interactions between the biological cell membrane and synthetic polymeric nanoparticles. Considering positively and negatively charged cores, the electrostatic repulsion between negatively charged cell membrane surfaces and polymeric PLGA could provide the right-side-out of the membrane orientation during the coating procedure. According to their results, the abundant and negatively charged sialyl residues from isolated membrane award a charge asymmetry across the membranes which can affect interfacial interactions with synthetic polymeric particles (Luk et al., 2014). Therefore, the desired coating process should be favored according to our results (Table 2).

### 4.2 Evaluation of isolated MB integrity and composition by mass spectrometry-based proteomics

Polyacrylamide gel electrophoresis is a commonly used technique for protein separation by size or charge and is also often used for protein quantification (Schägger, 2006; Brunelle and Green, 2014). SDS is a strong anionic detergent that denatures disulfide secondary and tertiary structures and imparts negative charges that allows them to run in the gel and correlates the proteins with a charge/mass ratio allowing their molecular weights to be estimated (Schägger, 2006; Brunelle and Green, 2014).

Supplementary Figure S1 shows samples that were further analyzed by LC-MS/MS: U251 whole cell (1–3), freshly isolated U251 cell membranes (4–6), and isolated cell membranes after 6-month storage at −80° (7–9). The SDS gels have shown that, despite the intensities, replicate samples have shown similar band patterns indicating similar protein content. All samples were applied to the gel at the same concentration and by comparison with the BSA standard. Following, a new calculation was performed to adjust protein concentration prior to the proteomic analysis.

**FIGURE 1:**
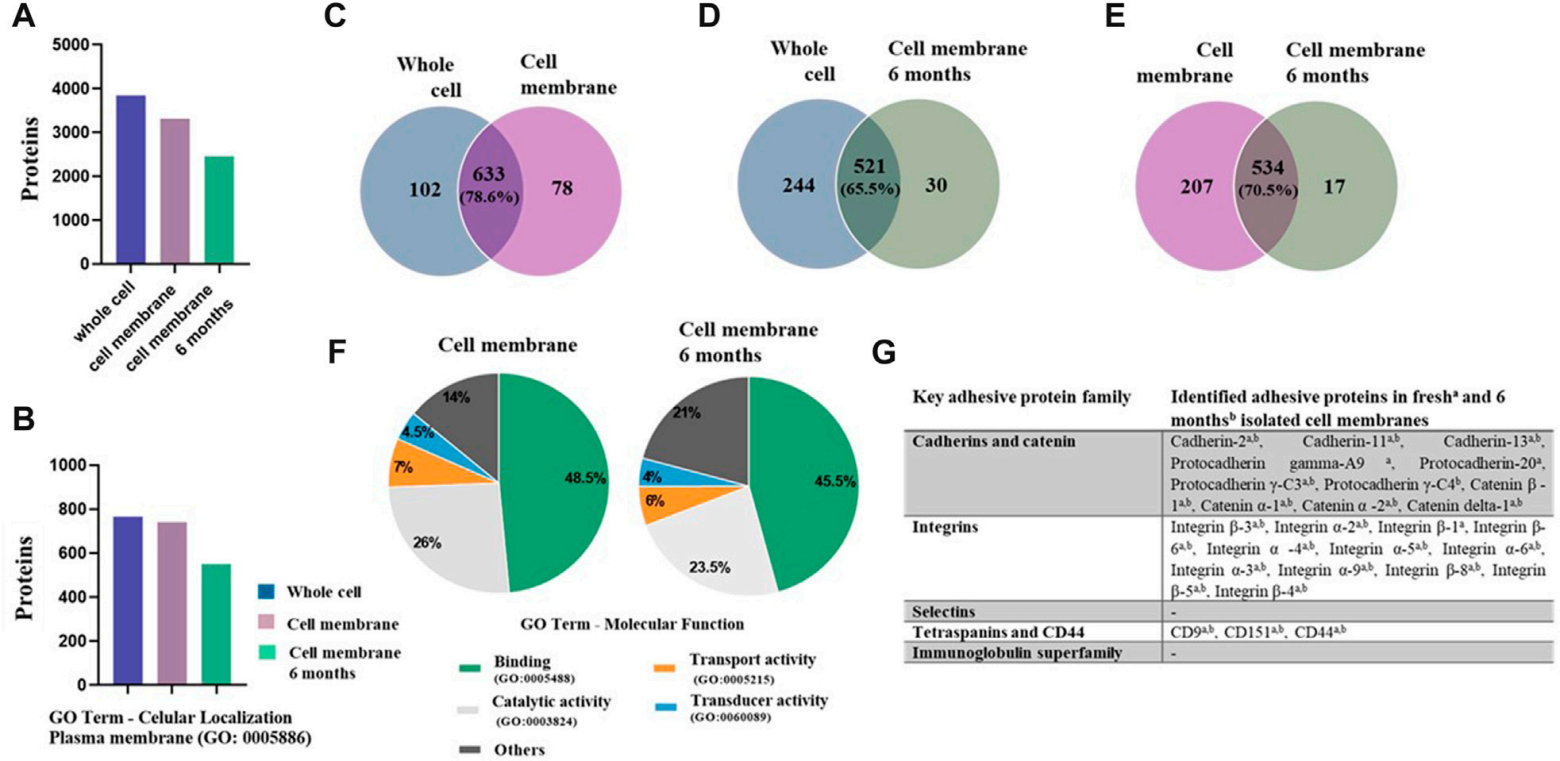
Qualitative data of proteomic analysis. Freshly isolated cell membranes, 6 months-stored isolated cell membranes, and whole U251 cells. **(A)** Total proteins quantified in freshly isolated cell MB, 6 months-stored isolated MB, and whole U251 cells. **(B)** Proteins were classified according to GO information. **(C–E)** Venn diagrams display profiles of cell membrane protein composition by overlapping each condition. **(F)** Cell membrane proteins were further classified according to their molecular function in freshly and 6 months-stored isolated cell membrane samples. **(G)** Key cancer cell adhesion proteins identified in freshly and 6 months-stored isolated cell membranes samples.

The biomimetic properties of NP-MB highly depends on the surface proteins retained by the isolated cell membranes, in particular, adhesion molecules such as cadherins, integrins, selectins, the immunoglobulin superfamily (Ig-SF), and homing receptors, which are critical for cell-cell and cell-matrix adhesion (Bose et al., 2018; Janiszewska et al., 2020). Therefore, a proper membrane proteome characterization is key to understand the homotypic binding effect. To gain further information regarding cell membrane protein composition, lable-free mass spectrometry-based proteomics analyses were conducted in freshly and 6 months-stored isolated cell MB, as well as in the whole U251 cells (Supplementary Figure S2). We were able to identify and quantify 3848 proteins in U251 cells, 3320 proteins in freshly isolated MB, and 2459 proteins in 6 months-stored isolated cell MB, in at least two out of three replicates (Figure 1A). Among these proteins, we were able to classify the ones associated with plasma membrane GO (0005886) in each group and found that 775 proteins of whole U251 cells proteome, 741 proteins of freshly isolated MB, and 551 proteins of 6 months-stored isolated cell MB were associated with this GO (Figure 1B). These results also showed that a large percentage of the identifications corresponds to non-cell membrane proteins, such as cytosolic, nuclear, and mitochondrial proteins, indicating that further membrane washing might be necessary to remove such contaminants.

Venn diagrams were built to examine the profiles of the cell membrane protein composition by overlapping each condition (Figures 1C–E). We observed that the freshly isolated cell MB shares nearly 80% of cell membrane proteins with the whole U251 cells; this result was considered positive as it means that we were able to retain almost 80% of these proteins after the membrane isolation process (Figure 1B). Upon comparing the proteome of whole U251 cells with fresh isolated MB, we observed that the number of retained-cell membrane proteins dropped to 70.5%. In addition, a comparison between the whole U251 cells and the 6-month isolated MB revealed that the retained-cell membrane proteins were 65.5% (Figures 1D, E, respectively).

**FIGURE 2:**
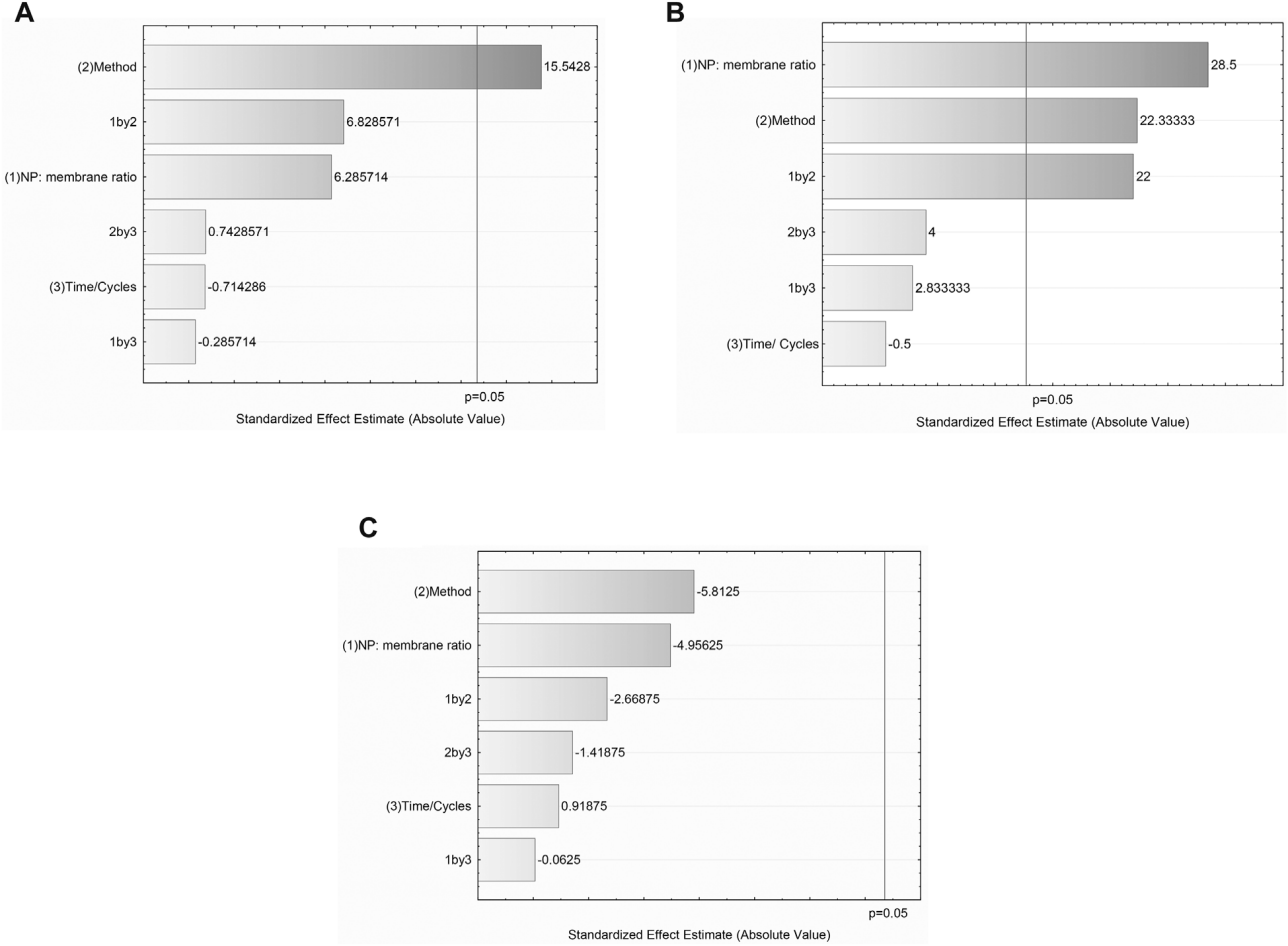
The Pareto charts of standardized effects ranked by their significance for any response. **(A)** Particle size **(B)** PDI, and **(C)** ZP. The dotted lines correspond to the *p*-value (0.05). Analyzed effects that are crossing this line are considered significant.

To investigate which proteins were kept in both MB samples, we further classified the cell membrane proteins into their molecular function, through GO analysis (Figure 1F). It is possible to observe that both samples display very similar profiles in terms of molecular function and that the GO “binding” represents almost 50% of proteins function for both groups. We have also manually searched the proteins belonging to the main adhesive protein families (Figure 2G). It is possible to observe that, although only 65% of membrane proteins were retained after 6-month storage in isolated MB (Figure 2D), most of the proteins responsible for the homotypic binding are present in these samples, suggesting that isolated MB can be stored for up to 6 months and keep its main adhesive proteins. In particular, the expression of Cadherin-2 is highly associated with cancer cells metastatic potential and cell-cell binding (Mrozik et al., 2018), therefore its presence in U251 isolated cell membrane (fresh and 6-month stored) might play a crutial role to bestow NP-MB homotipic binding properties.

### 4.3 Design of experiments (DoE) to optimize the development of membrane cell-derived hybrid nanostructures (NP-MB)

Several methodologies have been proposed to develop cell-derived hybrid nanostructures (Xiang et al., 2021). The physical extrusion, as the first method described and also largely applied, comprises the NP and isolated MB coextrusion through a porous membrane, where the disruption of membrane structure occurs by mechanical force and reorganization can be noticed around the polymeric core (Hu et al., 2011). More recently, the proposed bath sonication provided the spontaneous formation of membrane-coated nanostructures governed by ultrasonic energy (Copp et al., 2014).

Another variable quite diversified in the literature comprises exploring those systems in terms of the NP:membrane ratio applied for coating. Considering the latest publications regarding biomimetic membranes in PLGA nanoparticles, the use of the membrane-to-polymer ratio of 1:1 (particles) composes the most applied condition for preparation (Jin et al., 2019; Wang et al., 2020). Herein, based on those data and acquired results for mean particle size, the covering procedure will be further explored at 1:1 (w/w) and 1:5 (w/w). The required cycles in the case of physical extrusion and time for sonication bath should also be explored to optimize the procedure. Therefore, two widely used methodologies (physical extrusion and sonication bath), different NP: MB ratios applied for the coating process, and the time/cycles necessary for coating, were compared for NP-MB development in terms of size, PDI, and ZP results exploring statistical tools (Table 3).

**TABLE 3:** Codified variable composition of complete factorial design (2^3^) and acquired results regarding the optimization of membrane cell-derived hybrid nanostructures development.

Run order	Codified variables			Responses		
	NP: Membrane ratio	Method	Time/Cycles	Size (nm)	PDI	Zeta potential (Mv)
1	−1	−1	−1	164.7	0.157	−6.7
2	1	−1	−1	166	0.171	−11.9
3	−1	1	−1	180.4	0.149	−11.1
4	1	1	−1	202.1	0.289	−21.6
5	−1	−1	1	164.4	0.138	−4.5
6	1	−1	1	161.2	0.163	−6.7
7	−1	1	1	179.2	0.148	−10.2
8	1	1	1	203.4	0.311	−24.1
		NP		177 ± 30	0.13 ± 0.05	−1.98 ± 0.8
		MB		271 ± 8	0.5 ± 0.2	−23 ± 3.4
		NP/MB physical mixture		655 ± 52	0.9 ± 0.3	−19.3 ± 3

The applied variables, i.e., NP:membrane ratio, methodology, and time/cycles were optimized to acquire, as an indication of coverage, the slight increase in particle size together with changes in the PDI data (compared with controls). These variables were optimized by applying factorial design using StatSoft Inc. (2004) STATISTICA.

Initially, for comparative purposes, we performed the analysis of NP, MB, and physical mixture (MP: MB) in PBS under the same conditions as the DoE. Recorded values were 177, 271, and 655 nm for NP, MB, and physical mixture, respectively. The PDI data experienced an increase from NP to the physical mixture MP: MB, growing from 0.1 to a strongly polydisperse sample of 0.9. Recorded ZP values were negative, being greater for MB and physical mixture, evidencing the contribution of membranes negative ZP to this data.

DLS and NTA are the most accurate and reproducible methods to investigate the formation of the biological membrane coating. Different studies have reported hydrodynamic diameter values using the DLS technique to confirm the unilamellar membrane coating on the nanoparticulate core, since the increase in nanoparticle size may be related to the membrane coating (Hu et al., 2015a; Hu et al., 2015b). For the nanosystems produced here, although a negligible increase had been observed after the coating procedure with cell membranes, the increase in the ZP values of the NP was significant (Table 3) which contributed to the higher stability of the membrane-covered NP (NP-MB).

#### 4.3.1 The effect of applied variables on NP-MB particle size

The Pareto charts illustrated in Figure 2 show the standardized effects ranked by their significance on the critical attributes (particle size, PDI, and ZP). Particle size measurements of experimental conditions depicted NP-MB ranging from 161.2 to 203.4 nm (Table 3).

**FIGURE 3:**
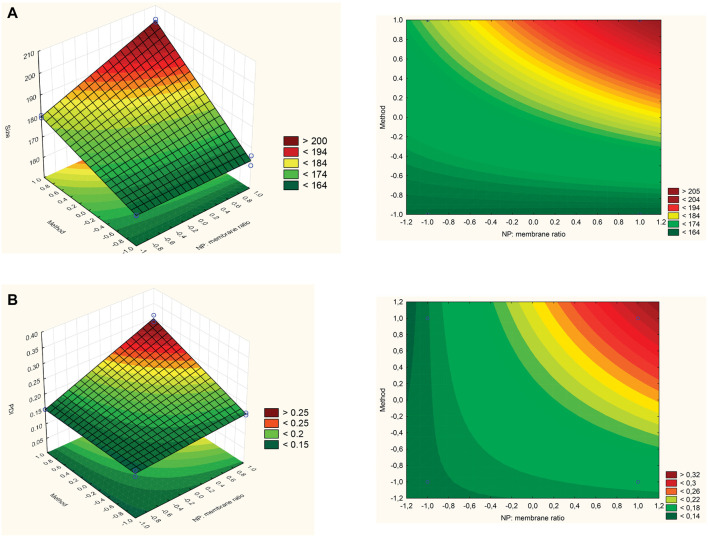
Response surface and contour plots. The effect of NP: membrane ratio and method on **(A)** nanostructures size and **(B)** nanostructures PDI.

According to the Pareto chart (Figure 2A), the adopted methodology (physical extrusion or sonication bath) had a significant effect on the NP-MB particle size, while the variable time has shown no significance to this attribute. The chosen method variable has shown a positive effect of 15.54, evidencing that when a sonication bath is applied for the coating procedure, NP-MB of larger size is produced. This effect can also be seen in the surface and contour graphs (Figure 3A), where the dark red area represents larger particles for samples that undergone sonication bath.

#### 4.3.2 The effect of applied variables on NP-MB polydispersity (PDI)

The PDI data, which varied between 0.138 and 0.311, strongly correlated with the NP:MB ratio and the adopted methodology *p* ≥ 0.05 (Figure 2B). Both analyzed variables produced a positive effect on the PDI increase (28.5 and 22.3), where even the interaction of these variables was significant (22.0). On the other hand, the variable time has shown no significance for PDI response. The contour graph evidenced dark red regions for high PDI values when the sonication bath and the NP:MB ratio of 1:5 are applied (Figure 3B). Therefore, the use of extrusion methodology allied with NP: MB 1:1 ratio, contributes to the acquisition of monodisperse nanostructures. This result was expected considering the contribution of PDI data related to the membrane analysis (MB PDI ∼0.5).

#### 4.3.3 The effect of applied variables on NP-MB zeta potential (ZP)

Considering the ZP results, the Pareto chart has shown that none of the studied variables had a significant influence on the ZP response. However, analyzing the ZP data of controls (NP, MB, and NP:MB physical mixture), we can observe that conditions 4 and 8 clearly show ZP values similar to those obtained in the physical mixture.

Taking these DoE data together, in a first step, we considered as an indication of coverage the slight increases on particle size together with changes in PDI data (compared with our control), and stability provided by the zeta potential value. In this regard, the use of the sonication bath procedure with NP:MB 1:1 ratio, as displayed in the run orders 3 and 7, seems to be the better condition to produce hybrid nanostructures in terms of size and homogeneity.

Since different run orders can be applied to the hybrid nanostructure development, further investigation on these conditions were evaluated using imaging techniques (negative-staining TEM) carrying out together with the periodic evaluation of size, PDI, and ZP for short-time stability.

### 4.4 Deeper physicochemical characterization of experimental conditions generated by DoE

**FIGURE 4:**
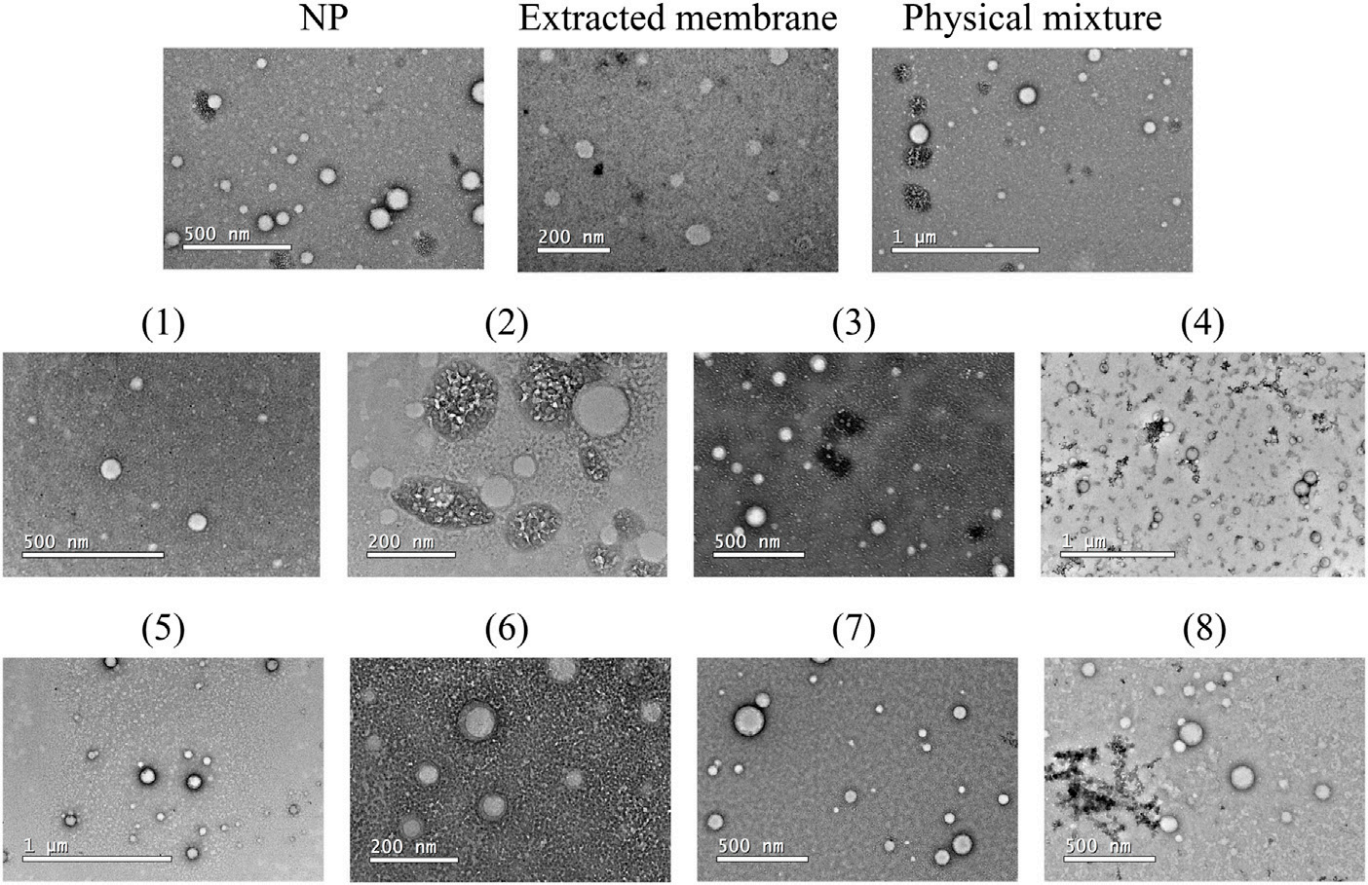
Morphological analysis of experimental conditions generated by DoE. Representative images of NP, MB (extracted membrane) as a negative control, and 8 run conditions by negative-staining TEM. Images were recorded using negative-staining transmission electron microscopy (TEM) JEM-2100-JEOL 200 with a LaB6 source operating at an acceleration voltage of 200 kV.

To shed light on the stability aspects concerning synthetic polymeric particles covered with natural cell membranes, the NP produced using the experimental conditions generated by DoE were morphologically characterized by negative-staining TEM, and their images are shown in Figure 4, which displays images of the negative controls in the first column (NP, extracted membrane, and physical mixture) and all experimental run conditions generated by DoE.

Negative-staining TEM has been used to characterize the core-shell structure of nanoparticles camouflaged with a biological membrane due to the difference in electronic densities between the outer cell membrane composed of lipids and proteins and the polymeric inner core (Kunde and Wairkar, 2021). The image analysis provided important conclusions that are not evidenced by the DoE. For example, it is noticeable that when the NP:membrane ratio 1:5 (w/w) was applied, the accumulation of organic matter can be easily visualized (run orders 2, 4, 6, and 8). The ZP analysis for these conditions did not allow this same conclusion because although the most negative values (closest to the MB control) were recorded when NP:membrane ratio 1:5 was applied, similar values were also observed for NP:membrane ratio 1:1.

The data generated by the DoE revealed that conditions 3 and 7 were the best ones to produce NP-MB nanostructures in terms of size and PDI. Considering the successful coating as revealed by the TEM images with different contrasts for the circular structures, we draw additional attention to conditions 6 that also clearly show this indication.

The general analysis of the data for short-time stability (2-week analysis) (Table 4) shows that there are no significant differences between the methodologies adopted in the production of NP-MB in terms of concentration (particles/mL), as evidenced by NTA. Following the same trend, no significant changes were observed in size and ZP from the initial analysis conditions and after 15 days. Overall, a small increase in PDI was observed in the second week, which may be associated with the stabilization of the surface groups. Importantly, the data still evidenced a monodisperse population for conditions 3 and 7.

**TABLE 4:** DLS parameters of short-time stability of membrane cell-derived coating nanostructures and NTA acquired data. DLS data are expressed as a mean of 3 different measures and standard deviation.

Run order	DLS analysis						NTA analysis		
	1 week			2 weeks			Size (nm)	Mode (nm)	Concentration particle x10^9^/mL
	Size (nm)	PDI	Zeta potential (Mv)	Size (nm)	PDI	Zeta potential (Mv)			
1	159 ± 4	0.11 ± 0.02	−4.5 ± 1.5	169 ± 15	0.23 ± 0.07	−4.8 ± 0.3	147	135	6.3 × 10^9
2	159 ± 2	0.12 ± 0.01	−8.4 ± 1.5	173 ± 7	0.20 ± 0.05	−8 ± 2	153	144	2.8 × 10^9
3	193 ± 21	0.15 ± 0.06	−6.9 ± 1.5	177 ± 3	0.20 ± 0.05	−9 ± 1	161	139	1.9 × 10^9
4	202 ± 7	0.27 ± 0.05	−18.6 ± 1.3	202 ± 8	0.30 ± 0.05	−16.8 ± 2.0	165	143	2.8 × 10^9
5	166 ± 4	0.15 ± 0.02	−3.6 ± 0.7	164 ± 7	0.20 ± 0.03	−3 ± 1	151	141	2.6 × 10^9
6	164 ± 9	0.12 ± 0.05	−7.4 ± 1.6	167 ± 8	0.20 ± 0.01	−3.3 ± 1.5	153	139	4.0 × 10^9
7	181 ± 9	0.19 ± 0.04	−8.8 ± 2.2	176 ± 6	0.20 ± 0.05	−6.8 ± 1.1	157	139	5.1 × 10^9
8	195 ± 11	0.23 ± 0.05	−16.7 ± 0.7	199 ± 6	0.30 ± 0.03	−17 ± 1	149	129	1.1 × 10^9

Therefore, considering both the data from DoE and the analysis of TEM images, and the evaluation of the short-term stability of these systems, we can conclude that the acquisition of an optimized NP-MB can be achieved for a NP:membrane ratio of 1:1 (w/w), sonication bath and 10 or 20 cycles of extrusion (condition contemplated in run 3 and 7). The images of the optimized formulations (Run 3 and Run 7) as recorded from DLS and the electrophoretic mobility experiments can be seen in Supplementary Figure S3. After NP-MB optimization, we have focused our attention on different physicochemical techniques to evidence the success of the coating procedure in the selected condition.

### 4.5 Vibrational spectroscopies and confocal characterization of optimized NP-MB nanostructure

**FIGURE 5:**
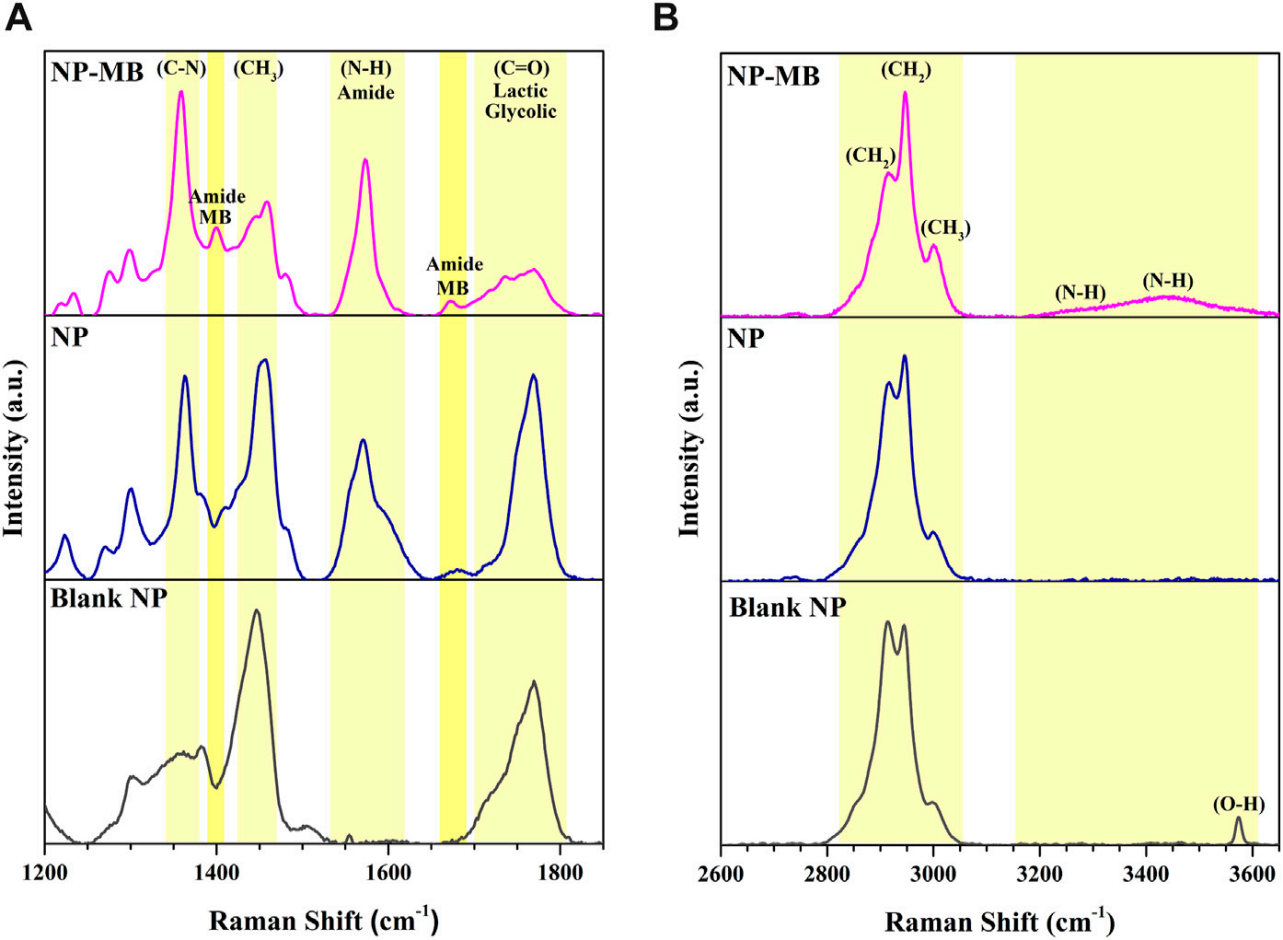
Raman spectra of Blank NP, NP and optimized NP-MB. **(A)** Raman shift from 1200 to 1900 cm^-1^. **(B)** Raman shift from 2600 to 3600 cm^-1^.

Raman spectra of the Blank NP, NP, and NP-MB (Figure 5) were obtained for comparative characterization and to check about physicochemical evidence of the coating procedure applying isolated U251 cell membrane. The Raman spectrum of Blank NP is mainly ascribed to vibrational modes of PLGA and PVA polymers (see Supplementary Figure S4). The band centered at 1450 cm^−1^ is assigned to the antisymmetric deformation mode of–CH_3_ groups from lactic units, whereas the band at 1768 cm^−1^ is related to stretching vibrations of C=O bonds from the lactic and/or glycolic polymeric units (Yan et al., 2015). The bands between 2800 and 3000 cm^−1^ are mainly attributed to the stretching modes of −CH_2_ (lactic unit) and −CH_3_ (glycolic unit) (Yan et al., 2015) from PLGA and the stretching vibration of −CH_2_ from PVA (Shi et al., 2017). All these bands, although sometimes in different intensities, could also be seen in the NP and NP-MB spectra.

As shown in Figure 5, the NP spectra exhibit additional bands related to the C-N stretch at 1358 cm^−1^ and N-H bend at 1578 cm^−1^ which can be assigned to the TMZ drug profile (Supplementary Figure S4) (Babu et al., 2012) reinforcing the drug encapsulation into the PLGA core. Additional signals were found in the NP-MB at 1400 cm^−1^ and at 1668 cm^−1^ which were assigned to the amide bands previously reported for the MB samples as typical amino acids from isolated glioma cell membrane protein (Pasquale et al., 2020). The latter was also evidence that the coating process successful occurred.

**FIGURE 6:**
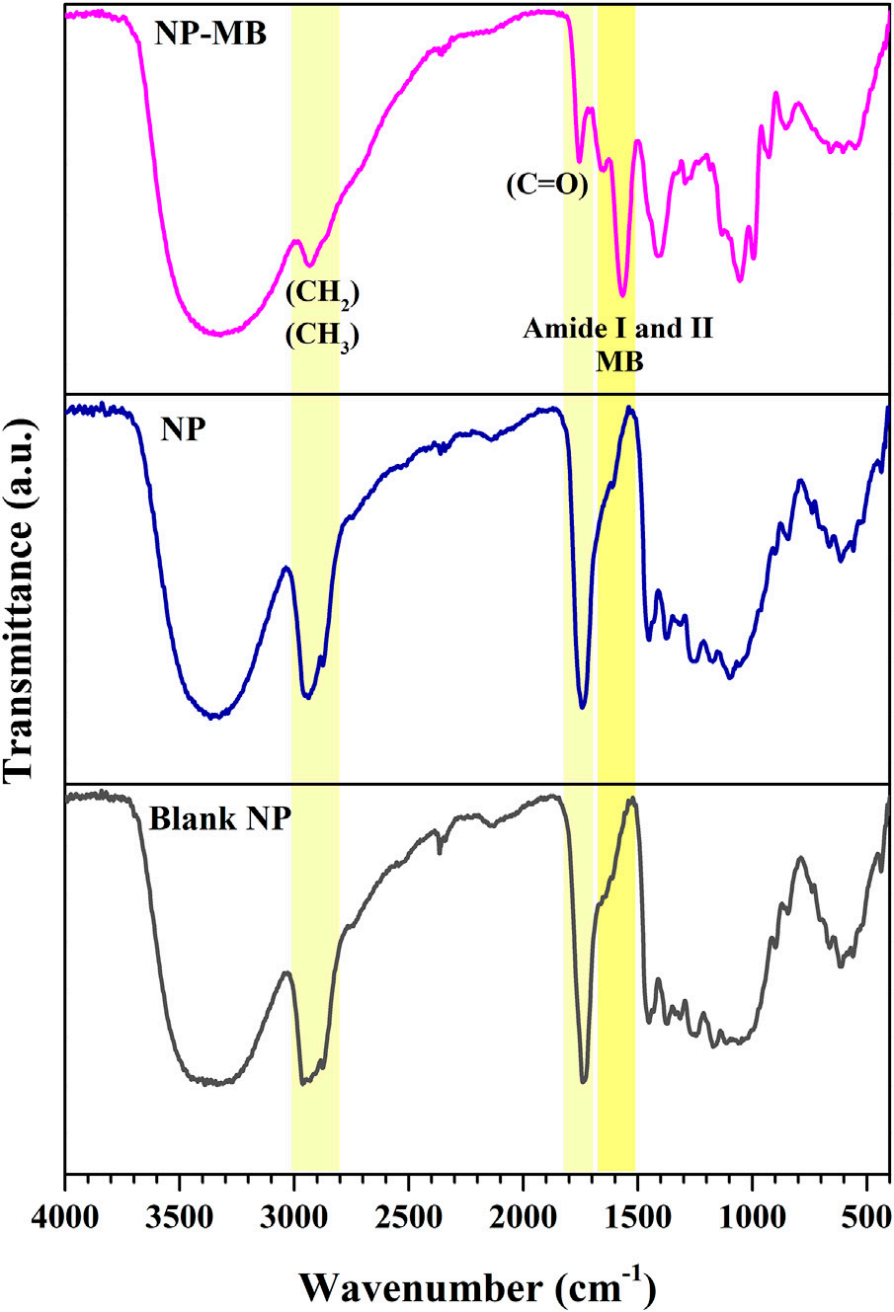
FTIR spectra of Blank NP, NP and optimized NP-MB.

The Raman analyses outputs were supported by the FTIR spectra of the same samples shown in Figure 6.

FTIR analysis allows the physicochemical characterization of the functional groups present in the drug, polymeric nanostructures, and isolated MB (Kadhim et al., 2022). Herein, the analysis was conducted for further evaluation of the molecular structure of NP constituents and to investigate possible chemical interactions between NP and TMZ drug and/or between NP and MB.

The FTIR spectra of the TMZ drug (Supplementary Figure S5) was similar to those reported previously (Irani et al., 2017), and shows typical bands around 3380 cm^−1^ due to the presence of N-H stretching. The asymmetric stretchings of alkyl groups and amide (-NH-C=O) can be observed at 3115 cm^−1^ and 1730 cm^−1^, respectively. The carbonyl stretching vibrations (C=O) is highly polar in nature and recorded as a strong and intense band at 1757 cm^−1^ (Wang et al., 2013; Ferreira et al., 2020). The NH_2_ scissoring vibration is observed in 1650–1620 cm^−1^. Additional signals at 1675 cm^−1^ and 1448 cm^−1^ are due to the N-H and C-H bending vibrations (Kadhim et al., 2022).

The spectrum of PLGA raw material (Supplementary Figure S5) has shown the typical antisymmetric stretching modes of -CH_2_ and -CH_3_ groups between 2900 cm^−1^ and 3000 cm^−1^, C=O stretching vibration at 1744 cm^−1^, the C–H stretching from methyl groups at around 1462 and C–O–C stretching mode at around 1080 cm^−1^ (Wang et al., 2013; Ferreira et al., 2020).

As shown in Figure 6, the FTIR spectra of blank NP, as well as NP exhibited the main modes observed in PLGA reference compound. Therefore, the spectra of these two samples are very similar and related to the PLGA composition. The presence of distinctive peaks directly associated with the TMZ drug interaction with the polymeric matrix has not been evidenced for the NP sample, as were observed *via* RAMAN spectroscopy. Therefore, considering both the data from Raman and FTIR analyses, TMZ molecules probably interacted with the polymer chains by supramolecular forces.

The MB sample exhibited a complex FTIR spectrum due to the absorption of several molecules, including lipids, and proteins (Mereghetti et al., 2014). As we can observe (Supplementary Figure S5), the spectrum of extracted MB exhibits the typical amide I and amide II bands between 1700 cm^−1^ and 1500 cm^−1^ as a result of the C=O stretching and the NH bending from peptides, respectively, which is also observed in NP-MB sample (Figure 6). The coating procedure using biological membranes is not yet fully understood, and should rely on a simple non-covalent hydrophobic interaction between lipids and protein from isolated MB, that favors the formation of the coating around NP to minimize unfavorable interactions with the aqueous solvent (Pasquale et al., 2020). Hence, these additional bands in NP-MB spectra and which not coincide with those observed in TMZ reference or in blank NP and NP samples, may support the presence of an external MB organic coating.

**FIGURE 7:**
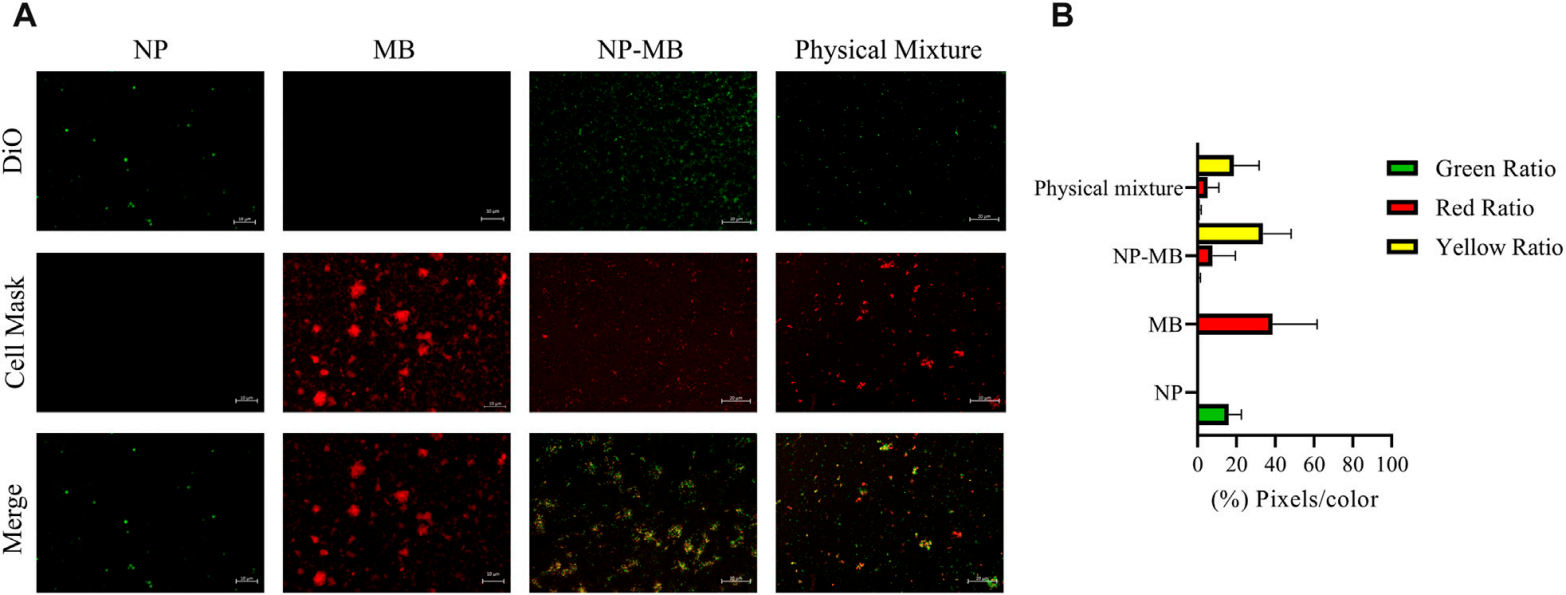
Confocal microscopy images recorded from NP, MB, NP-MB, and physical mixture (NP + MB) to identify visual evidence of the coating process. **(A)** Representative images recorded from NP, MB, NP-MB, and physical mixture for data processing. Green represented the fluorescence of DiO dye (λem = 521 nm) and Red represented the fluorescence of CellMask™ Deep Red dye (λem = 670 nm). Scale bars = 20 μm. **(B)** Data processing using a python programming language with OpenCV library for sample categorization.

To further investigate the coating procedure, NP and MB samples were doped with DiO and Cell MaskTM dyes and subjected to the optimized specific condition to produce NP-MB. Images recorded from NP, MB, NP-MB, and the physical mixture were exposed to the same brightness and contrast adjustments and are representative of the experiment done in replicates (n = 5) (Figure 7A).

As revealed in Figure 7A, for NP samples stained with DiO, the recovery of fluorescence at 521 nm was recorded while no fluorescence signal could be observed at 670 nm. The merged image of those samples only exhibited a green signal. On the other hand, MB sample have shown no signal at 521 nm but intensive red fluorescence at 670 nm as well as merged image. For NP-MB, physical mixture, yellow color represents significant colocalization of fluorescent signals observed at the merge image. Importantly, the NP-MB appears to show more intensive fluorescent signals related to colocalization areas than physical mixture since they have shown more well-defined green and red isolated dots. Our results were similar to data reported by previous papers regarding the coating using natural cell membranes (Jiang et al., 2019; Nanobiotechnol et al., 2020; Li et al., 2021). These results may indicated the successful fusion of natural cell membranes for NP-MB. The further quantification of these features was carried out by using a python programming language with OpenCV library. As a result, a greater % of yellow pixels for NP-MB (34%) was recorded when compared with the registered % for the physical mixture (19%) evidencing that the colocalization index tends to be higher for NP-MB (Figure 7B).

Taken together, RAMAN, FTIR spectroscopy, and confocal images strongly support the encapsulation of TMZ drug into the polymeric PLGA core and also the successful procedure using the coating of nanosystems with isolated tumor cell membrane MB.

### 4.6 Internalization studies and evaluation of homotypic targeting ability of optimized MB-NP nanostructure

Biomimetic functionalization of drug delivery nanostructures with isolated cancer cell membranes confers great advantages as it increases biocompatibility, due to its immune system evading properties, in addition to allowing the homotypic binding (Sun et al., 2016; Bose et al., 2018). This mechanism comprises the affinity that cancer cells have to bind to each other, due to the presence of specific cell-cell adhesion proteins on its surface (Fang et al., 2014; Zhu et al., 2016). Consequently, the cancer cell membrane-coated NP shows great specificity/targetability to the tumor source cells, as demonstrated by several reports in literature (Mohammad et al., 2020; Han et al., 2021; Zhang et al., 2022).

**FIGURE 8:**
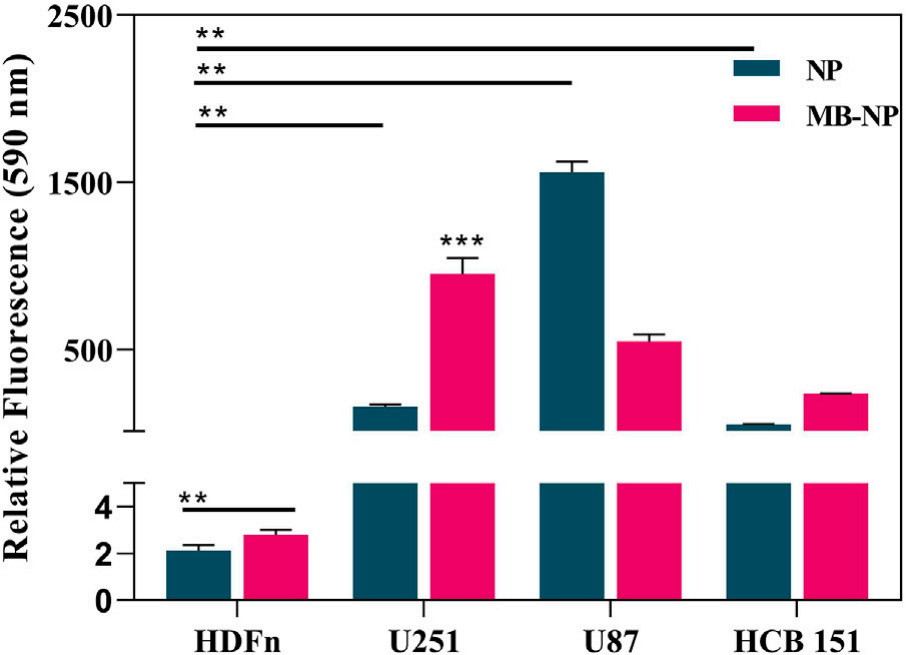
Evaluation of homotypic targeting ability of NP-MB. Cell internalization profile of NP and MB-NP in non-tumoral HDFn and different glioma cell lines. HDFn, U251, U87, and HCB 151 cells were incubated for 8 h with DiO-labeled NP and MB-NP and processed for flow cytometry analysis, by which the fluorescent intensities of both nanostructures were acquired. The results express the geometric mean of the fluorescence intensity divided by the average of a blank cell. Data represent the mean ± SD of three independent replicates (n = 3).

Here, the homotypic binding was observed after incubating HDFn (non-tumoral cell) U251, U87, and HCB151 cells with fluorescent-labeled NP and MB-NP (Figure 8). The biomimetic nanostructure was built with a U251 isolated cell membrane and, as expected, showed greater affinity to U251 cells than to the other GBM cell lines. The internalization of MB-NP in U251 cells was almost 2 times greater compared to U87 cells, and nearly 4 times greater compared to HCB151 cells. Most importantly, the internalization of both NP and NP-MB was much lower for HDFn (non-tumoral cell) than for all tumoral cells.

In addition, for both U251 and HCB151 cells, the nanostructure internalization greatly benefited from the cancer cell membrane coating and homotypic binding. Interestingly, the opposite was observed for U87 cells. In this case, non-coated NP was more easily internalized than MB-NP.

Although these cells are classified as glioblastomas, one of the main characteristics of this type of cancer is the high tumor heterogenicity (Friedmann-Morvinski, 2014), which may explain the different internalization patterns among them. It is possible that not only significant differences in the cell-cell adhesion proteins between these cells were a key factor determining the lower MB-NP uptake by U87 and HCB151 cells, but also factors such as their uptake kinetics.

## 5 Conclusions

In conclusion, we have reported the synthesis optimization for bioinspired and biomimetic nanostructures composed of PLGA polymeric core loaded with the TMZ drug, further coated with extracted U251 cell membrane. Important knowledge regarding extracted MB stability and recovery aspects was achieved using mass spectrometry-based proteomics. The optimization of the biomimetic nanostructured system was achieved by applying, for the first time, the statistical tool design of the experiment together with physicochemical, TEM analyses, and evaluation of short-time stability. The associated techniques provided the ideal structure for obtaining the desired properties, also providing evidences of the successful NP coating by different techniques. The results of cell internalization indicated that the optimized NP-MB achieved specific targeting of homotypic tumor cells. Given the evidence of the coating and homotypic binding of the source tumor cells and the decrease of the internalization of such structure in non-tumoral cell models, the biomimetic nanoparticles developed and optimized here can serve as a smart nanoplatform for cancer treatments based upon homotypic targeting.

## Data Availability

The data presented in the study are deposited in the ProteomeXchange via the PRIDE database repository, accession number PXD039270.
