# Supplementary material for: Using design of experiments (DoE) to optimize performance and stability of biomimetic cell membrane-coated nanostructures for cancer therapy
Source: Front Bioeng Biotechnol. 2023 Feb 2;11:1120179. doi: 10.3389/fbioe.2023.1120179 (PMC9932601; doi:10.3389/fbioe.2023.1120179)
Supplement: Supplementary file 1 [file DataSheet1.docx]

**USING DESIGN OF EXPERIMENTS (DoE) TO OPTIMIZE PERFORMANCE AND STABILITY OF BIOMIMETIC CELL MEMBRANE-COATED NANOSTRUCTURES FOR CANCER THERAPY**

Natália Noronha Ferreira^1^†**^**^**; Renata Rank Miranda^1^†; Natália Sanchez Moreno^1^; Paula Maria Pincela Lins^1^; Celisnolia Morais Leite^1^, Ana Elisa Tognoli Leite^1^, Thales Rafael Machado^1^; Thaís Regiani Cataldi^2^, Carlos Alberto Labate^2^, Rui Manuel Reis^3,4,5^ and Valtencir Zucolotto^1^**.**

^1^Nanomedicine and Nanotoxicology Group, *Physics Institute of São Carlos, São Paulo University*. Avenida Trabalhador São Carlense, 400, CEP13560970 (Brazil) Phone: (+55) 16 3373 8656*, São Carlos – SP – Brazil.*  natalia.noronha@usp.br ORCID: 0000-0002-5090-9971; renatam.bio@gmail.com ORCID: 0000-0001-8774-2418; ppincela@gmail.com ORCID: 0000-0002-8663-4463; [natalia.smoreno@gmail.com](mailto:natalia.smoreno@gmail.com) ORCID: 0000-0001-9276-1939; [natognoli@hotmail.com](mailto:natognoli@hotmail.com) ORCID: 0000-0002-4300-9414; [celisnolia@hotmail.com](mailto:celisnolia@hotmail.com) ORCID: 0000-0003-0933-9767; tmachado.quimica@gmail.com ORCID:0000-0002-3246-6329; [zuco@ifsc.usp.br](mailto:zuco@ifsc.usp.br) ORCID: **0000-0003-4307-3077**

^2^Max Feffer Laboratory of Plant Genetics, Department of Genetics, ESALQ, University of São Paulo, Av. Pádua Dias 11, 13418-900, Piracicaba, São Paulo, Brazil. thais.cataldi@usp.br; ORCID 0000-0002-9827-2017; [calabate@usp.br](mailto:calabate@usp.br) ; ORCID 0000-0001-7309-1300.

^3^Molecular Oncology Research Center, Barretos Cancer Hospital, Barretos, SP, Brazil. ruireis.hcb@gmail.com; ORCID 0000-0002-9639-7940.

^4^Life and Health Sciences Research Institute (ICVS), School of Medicine, University of Minho, 4704553 Braga, Portugal; [rreis@med.uminho.pt](mailto:rreis@med.uminho.pt); ORCID 0000-0002-9639-7940

^5^ICVS/3B’s—PT Government Associate Laboratory, 4704553 Braga, Portugal; [rreis@med.uminho.pt](mailto:rreis@med.uminho.pt); ORCID 0000-0002-9639-7940

*______________________________________________________________________*

****CORRESPONDING AUTHOR:**

Dr. Valtencir Zucolotto

Avenida Trabalhador São Carlense, 400, CEP13560970 (Brazil) Phone: (+55) 16 3373 8656*, São Carlos – SP – Brazil.*  email: zuco@ifsc.usp.br.

**SUPPLEMENTARY MATERIAL (SM)**


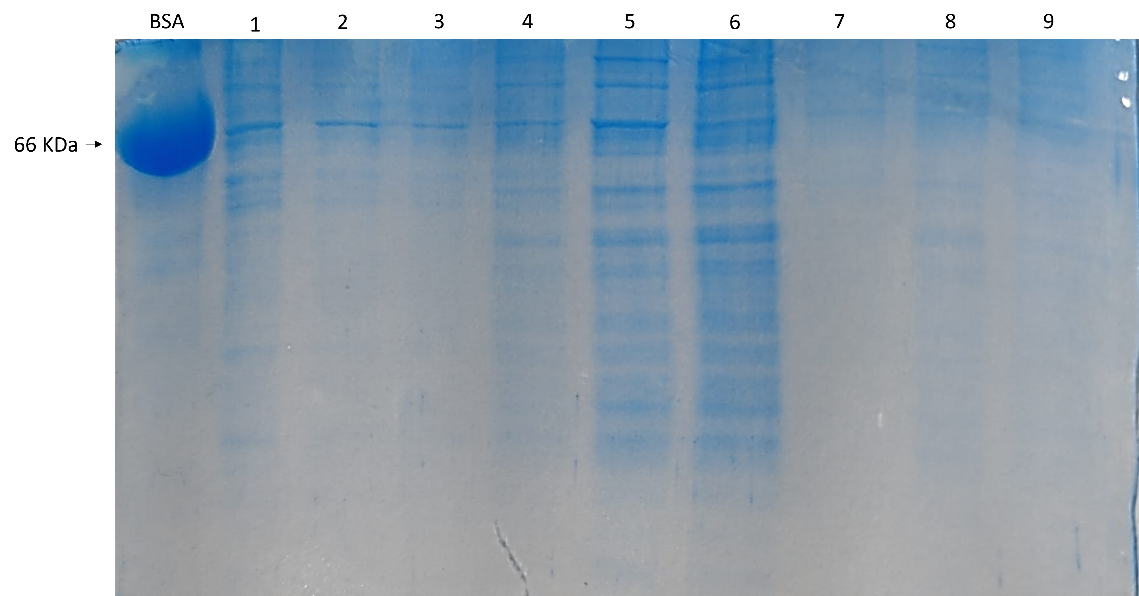


**Figure SM1:** Samples analyzed by mass spectrometry chromatography-tandem MS: U251 whole cell (1 to 3), freshly isolated U251 cell membranes (4 to 6), and isolated cell membranes after 6-month storage at -80º C (7 to 9). All samples were applied at the same concentration and by comparison with the BSA standard, a new calculation for adjusted protein concentration was performed prior to the proteomic analysis.

**Figure SM2: (a)** PCA analysis of cellular proteins identified and quantified in whole cell proteome and freshly isolated cell membranes. **(b)** Volcano plot of freshly isolated cell membranes in relation to whole cell proteome. Important features selected by volcano plot with fold change threshold (x) 4 and t-tests threshold (y) 0.05. The red dots represent features above the threshold and blue dots represent features below the threshold. **(c)** PCA analysis of cellular proteins identified and quantified in 6-month isolated cell membranes and freshly isolated cell membranes. **(d)** Volcano plot of 6-month isolated cell membranes in relation to freshly isolated cell membranes. Important features selected by volcano plot with fold change threshold (x) 4 and t-tests threshold (y) 0.05. The red dots represent features above the threshold and blue dots represent features below the threshold.

| **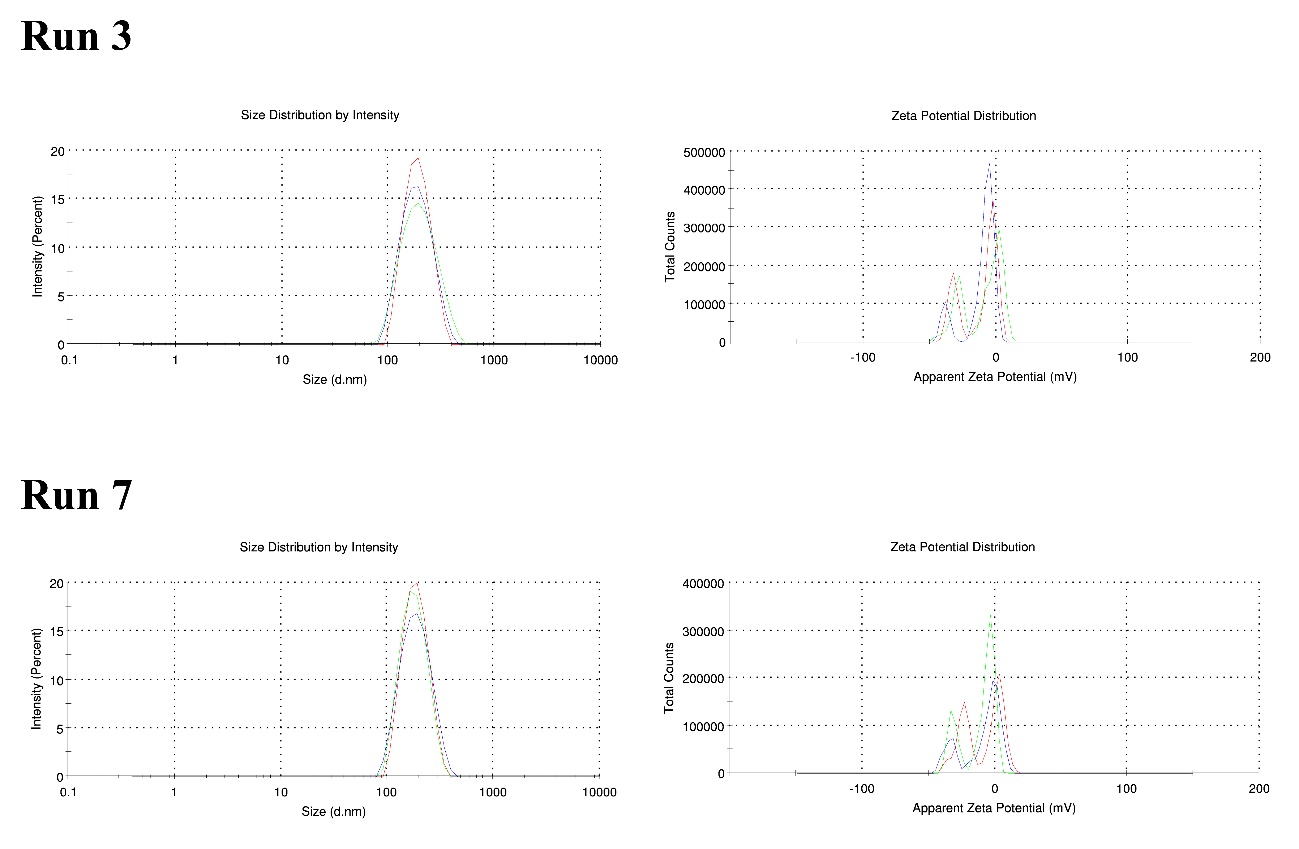** |
| --- |

**Figure SM3:** Images of the optimized formulations Run 3 and Run 7. Particle size and zeta potential recorded from Dynamic Light Scattering (DLS) and the electrophoretic mobility in a Zetasizer Nano ZS (Malvern Instruments, Malvern, UK) equipment.

| **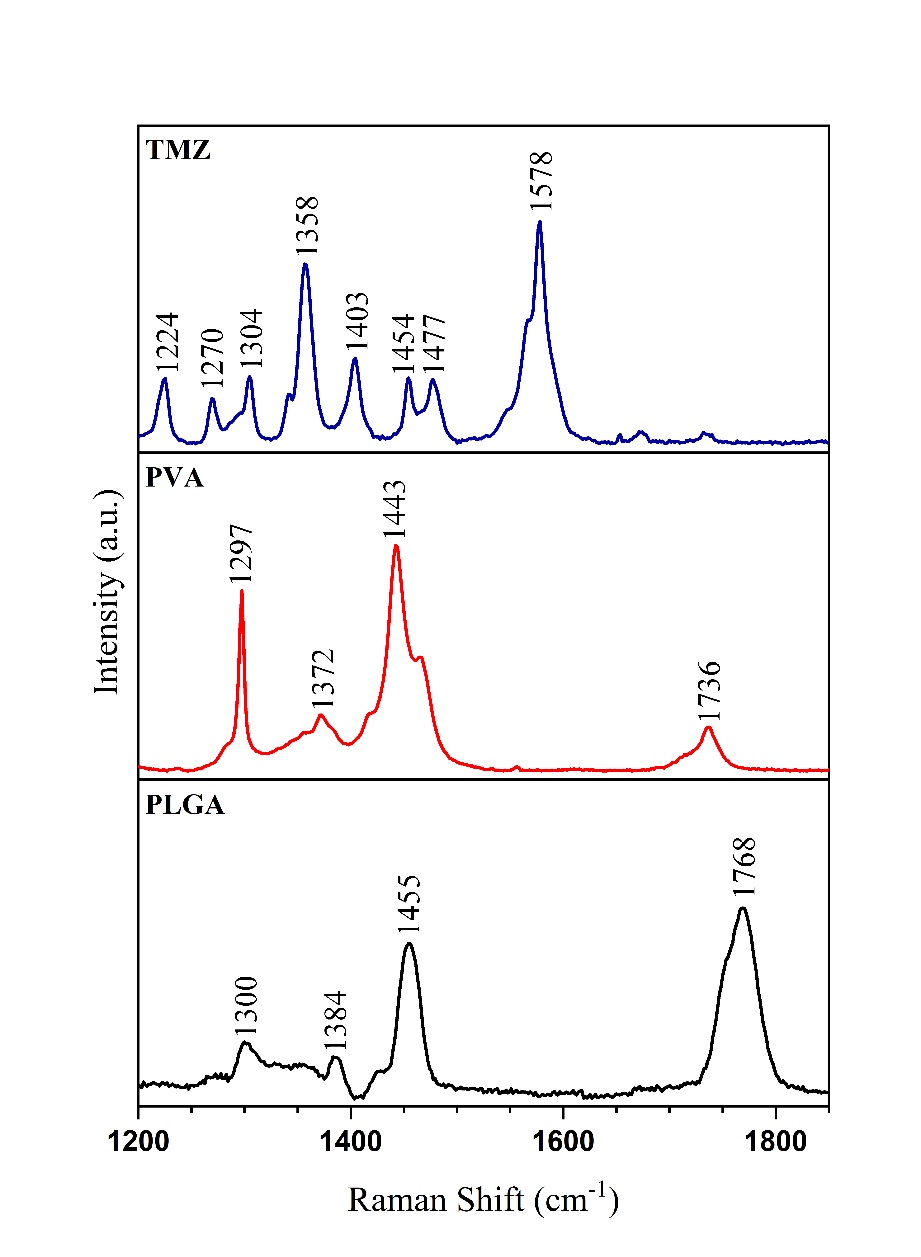** |
| --- |

**Figure SM4:** RAMAN spectra of the isolated materials to produce NP and NP-MB. PLGA only, in black; the PVA solution in red, and the TMZ drug in blue. The spectra are baseline-corrected.

| **** |
| --- |
| **** |

**Figure SM5:** FTIR spectra of the isolated materials to produce NP-MB.
